# Linear and nonlinear characteristics of the runoff response to regional climate factors in the Qira River basin, Xinjiang, Northwest China

**DOI:** 10.7717/peerj.1104

**Published:** 2015-07-21

**Authors:** Jie Xue, Dongwei Gui

**Affiliations:** 1 State Key Laboratory of Desert and Oasis Ecology, Xinjiang Institute of Ecology and Geography, Chinese Academy of Sciences, Xinjiang, China; 2 Cele National Station of Observation and Research for Desert-Grassland Ecosystem, Xinjiang Institute of Ecology and Geography, Chinese Academy of Sciences, Xinjiang, China; 3 Key Laboratory of Biogeography and Bioresource in Arid Land, Xinjiang Institute of Ecology and Geography, Chinese Academy of Sciences, Xinjiang, China; 4 University of Chinese Academy of Sciences, Beijing, China

**Keywords:** Climate change, Runoff response, Qira River basin, Linear characteristics, Nonlinear characteristics, Oasis, Xinjiang

## Abstract

The inland river watersheds of arid Northwest China represent an example of how, in recent times, climatic warming has increased the complexity of Earth’s hydrological processes. In the present study, the linear and nonlinear characteristics of the runoff response to temperature and precipitation were investigated in the Qira River basin, located on the northern slope of the Kunlun Mountains. The results showed that average temperature on annual and seasonal scales has displayed a significantly increasing trend, but this has not been reflected in accumulated precipitation and runoff. Using path analysis, a positive link between precipitation and runoff was found both annually and in the summer season. Conversely, it was found that the impact of temperature on runoff has been negative since the 1960s, attributable to higher evaporation and infiltration in the Qira River basin. Over the past 50 years, abrupt changes in annual temperature, precipitation and runoff occurred in 1997, 1987 and 1995, respectively. Combined with analysis using the correlation dimension method, it was found that the temperature, precipitation and runoff, both annually and seasonally, possessed chaotic dynamic characteristics, implying that complex hydro-climatic processes must be introduced into other variables within models to describe the dynamics. In addition, as determined via rescaled range analysis, a consistent annual and seasonal decreasing trend in runoff under increasing temperature and precipitation conditions in the future should be taken into account. This work may provide a theoretical perspective that can be applied to the proper use and management of oasis water resources in the lower reaches of river basins like that of the Qira River.

## Introduction

Water is an extremely important natural resource for the sustainable social and economic development of semiarid and arid regions. Consequently, the ecological environment is highly vulnerable and suffers from severe threats due to the scarcity and excessive utilization of water resources (Boehmer, Memon & Mitchell, 2000; Chen & Xu, 2005; Ling et al., 2011). Xinjiang, Northwest China, is one of the world’s largest arid areas, characterized by seriously fragile water resources and associated ecological and environmental challenges (Chen, 2014). The landscape is a typical mountain–oasis–desert ecosystem. The rivers generally originate from the mountainous regions because the precipitation there, which exists as snowfall, is temporarily stored as snowpack and/or ice cover, while the precipitation that occurs in the oasis and desert areas is unable to form effective surface runoff (Li et al., 2011b). The oases, situated between the mountainous areas and amongst the desert plains, essential for human settlement and combating desertification (Ling, Xu & Fu, 2013), require a stable water supply. In recent years, however, the increased water demands related to human activities, such as agricultural irrigation and oasis extension in the lower reaches of the river basin, have become a significant challenge for the sustainable development of water resources in such arid regions.

The formation of surface runoff, an important component of water resources, is a complicated hydro-climatic process, and its response to climate change is quite sensitive in the inland river basin in Xinjiang (Li et al., 2011a; Chen, 2014). Indeed, climatic variation has been shown to significantly impact regional or local watershed hydrological processes (Liu et al., 2009; Ling et al., 2011). Specifically, it is widely accepted that the climate of Xinjiang has begun to transform from one of warm and dry conditions to warm and wet conditions (Shi et al., 2007; Li et al., 2012). Regional- or watershed-scale hydrological processes and cycles are inevitably impacted by this remarkable change.

Coupled climatic and hydrological processes involve complex linear and nonlinear mechanisms and characteristics at both the regional and watershed level (Kantelhardt et al., 2003; Xu et al., 2009; Xu et al., 2010). Hydrological models have been developed to reproduce the runoff process generated by climatic and other variables. The use of such models can assist in deepening our understanding of physical hydro-climatic mechanisms. However, long-term climatic changes can alter the runoff generation process, and thus the timing and frequency of hydrological events. It is difficult to clarify the linearity and nonlinearity of the hydrological response to climate change over longer time scales based on the two main meteorological–hydrological model approaches, both of which have their drawbacks: Black-box models, such as neural network models, cover up the interactive relationship between climatic variables and runoff; while watershed hydrology models, through their representation of the underlying physical mechanisms, do integrate the effects of various factors, such as soil and vegetation, but not without uncertainty.

The Qira River is a typical inland river in the southern rim of the Tarim basin. Similar to other rivers in this region, its runoff consists mainly of glacial/snowmelt water and precipitation. With the intensification of climate change, the increasing population and agricultural irrigation, and especially the recent extension of the oases in the Qira River basin, the river runoff has gradually decreased and the impacts on the eco-environmental system have severely worsened (Dai et al., 2009a; Dai et al., 2009b). The magnitude of climate change locally, together with the increased water demands in this severely moisture-deficient basin, has led to the occurrence of many droughts in the lower reaches of the Qira River. Meanwhile, extreme weather variability also induces severe hydrological events, at both ends of the spectrum, i.e., droughts and floods. For example, in late July and early August 2010, the Qira River experienced its heaviest flood on record since 1961. The economic cost of the damage (large areas of submerged farmland and villages) was huge. The shift from a warm and dry climate to a warm and humid climate shows that climatic factors (e.g., temperature and precipitation) have led to a significant change in the runoff of the Qira River, as well as others in the southern rim of the Tarim basin. Thus, there is an urgent need to investigate the runoff response to long-term climate change in the Qira River basin, as this will enable the better use and management of the water resources and oases in this region, as well as assist planners and decision makers to find the right measures for the further development of the region against the background of climate change.

Although many studies have attempted to explore the mechanisms and characteristics of hydro-climatic processes, their linearity and nonlinearity has been less well considered, meaning a detailed understanding of these processes and their interactions remains limited. In particular, few attempts have been made to identify the key characteristics of the relationships between climatic factors and runoff in the long-term time series of alpine areas. In the Qira River basin specifically, only short-term observational time series from Kartash Station (2,800 m above sea level), located in the upstream region, have been used to investigate the meteorological characteristics (Osamu & Wang, 2004). Furthermore, while the changing trend and features of runoff in the Qira River have been analyzed (Dai et al., 2009a; Dai et al., 2009b), the impact of long-term climate change on runoff in the Qira River basin has attracted less attention. Therefore, in the present study, using the Qira River basin as the study area, the characteristics of hydro-climatic processes were explored from a new perspective and by using appropriate methods.

Specifically, based on data from meteorological and hydrological stations within the Qira River basin, statistical methods including regression analysis, the Mann–Kendall trend test, and path analysis were used to analyze the linear characteristics of meteorological–hydrological processes. In addition, the Mann–Whitney abrupt-change test, wavelet analysis, correlation dimension, and rescaled range (*R*/*S*) analysis were applied to investigate the nonlinear characteristics of the relationships between climatic factors and runoff in the Qira River basin. This study may not only provide a theoretical basis for the impact of regional climate change on runoff, but is also expected to act as a useful reference for other areas, especially those in the southern rim of the Tarim basin.

## Materials and Methods

### Research area and data

**Figure 1:**
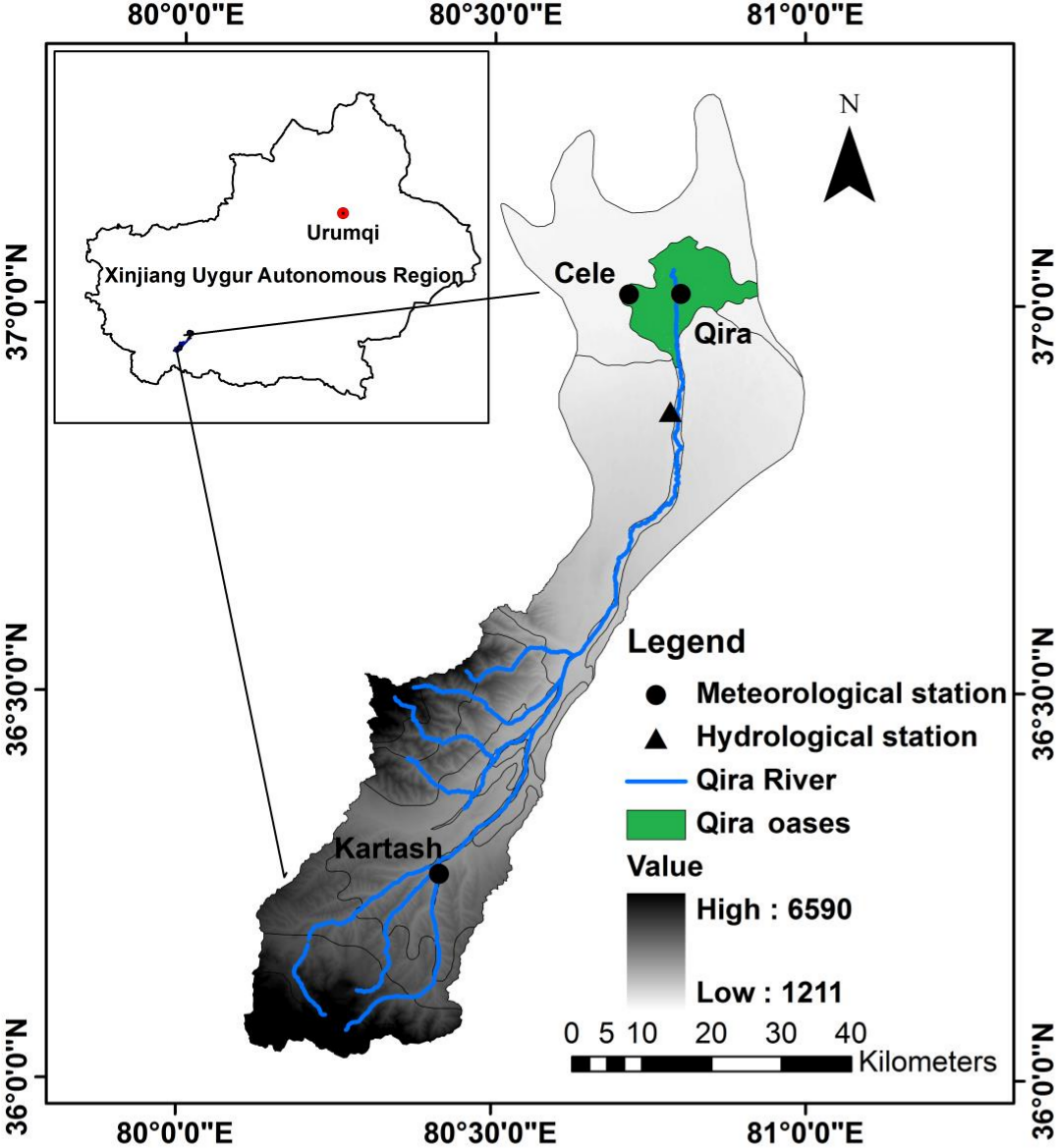
Location of the study area and stations.

The Qira River basin is located on the northern slope of the Kunlun Mountains and the southern rim of the Tarim basin in Xinjiang (36°02′–37°16′N, 80°07′–81°00′E), covering a basin area of approximately 3,328.51 km^2^ (Fig. 1). The Qira River is a dissipative inland river of about 136.2 km in length. Its source is in the high-altitude valleys of the Kunlun Mountains, from where it flows through the Qira oases, and finally discharges into the extremely arid desert. The unique landscape and ecosystem of the Qira River basin means that it is sensitive and vulnerable to climate variability (Zhou et al., 2006). Abundant precipitation and very low temperatures are a feature in the high-altitude regions (Wu, Yang & Hua, 2011; Wu et al., 2013). The glacier/snowpack is generally distributed at the top of the mountain range, grassland and shrubland characterize the middle-altitude areas, and oases are situated at its foot. The desert plains are then adjoined to the oasis areas (Wu, Yang & Hua, 2011; Wu et al., 2013).

The availability of station data in the basin is poor. There is only one station (Kartash) that provides long-term meteorological data, located in the upstream area of the river ((80°25′N, 36°16′E); 2,800 m above sea level), and one hydrological station ((80°48′N, 36°52′E); 1,557 m above sea level), which is 27 km away from the mountainous region. Unfortunately, the meteorological stations at Cele and Qira are situated downstream of the hydrological station. The station at Kartash, while at high altitude, offers insight into the climatic conditions of the basin, assuming that the observed meteorological records there are broadly representative. Therefore, to further investigate the interactive relationship between climate factors (temperature and precipitation) and runoff, this study used the monthly mean temperature and monthly accumulated precipitation from 1961 to 2010 at Kartash meteorological station, and the runoff recorded at the hydrological station.

### Methods

To explore the linear and nonlinear characteristics of the relationships between climate factors (temperature and precipitation) and runoff, this study applied the Mann–Kendall trend test, and path analysis to analyze the linear changes and significant tendencies between them, and the direct and indirect impacts of temperature and precipitation on runoff. In addition, the Mann–Whitney test, wavelet analysis, the correlation dimension method, and *R*/*S* analysis were respectively employed to reveal the abrupt changes, periodicities, chaotic dynamic characteristics, and long-term memory characteristics between temperature, precipitation and runoff in the Qira River basin.

#### Mann–Kendall trend test

The nonparametric Mann–Kendall trend test (Kendall, 1975; Mann, 1945) has been widely used to detect change trends in meteorological and hydrological time series (Burn, 2008; Zhang et al., 2010; Xu et al., 2013). The rank-based Mann–Kendall method was used in this study to test for significant tendencies in the temperature, precipitation and runoff time series in the Qira River basin.

In the Mann–Kendall trend test, the null hypothesis (*H*_0_) is that the parameter time series (*X_i_*, *i* = 1, 2, …, *n*) are independent and identically distributed random variables, while the alternative hypothesis (*H*_1_) is that there is a tendency in the variable time series (Partal & Kahya, 2006; Li et al., 2010). The standardized statistic of the Mann–Kendall trend test, *Z*, is defined by:

(1) \begin{eqnarray*} \displaystyle Z=\left\{\begin{array}{ll} \displaystyle \frac{S-1}{\sqrt{v a r(S)}},&\displaystyle S> 0\\ \displaystyle 0,&\displaystyle S=0\\ \displaystyle \frac{S+1}{\sqrt{v a r(S)}},&\displaystyle S\lt 0 \end{array}\right.&&\displaystyle \end{eqnarray*}

where

(2) \begin{eqnarray*} \displaystyle S=\sum _{i=1}^{n-1}\sum _{k=i+1}^{n}s g n({x}_{k}-{x}_{i}),&&\displaystyle \end{eqnarray*}

in which *x_k_* and *x_i_* are the series of temperature, precipitation and runoff; *n* is the length of the time series; *k* = 1, 2, …, *n* − 1, *j* = 2, 3, …, *n* and

(3) \begin{eqnarray*} \displaystyle s g n({x}_{k}-{x}_{i})=\left\{\begin{array}{ll} \displaystyle 1,&\displaystyle {x}_{k}-{x}_{i}> 0\\ \displaystyle 0,&\displaystyle {x}_{k}-{x}_{i}=0\\ \displaystyle -1,&\displaystyle {x}_{k}-{x}_{i}\lt 0. \end{array}\right.&&\displaystyle \end{eqnarray*}

Moreover, *S* approximately follows the normal distribution, with 0 mean, and the variance is given as:

(4) \begin{eqnarray*} \displaystyle v a r(S)=\frac{n(n-1)(2 n+5)}{18}.&&\displaystyle \end{eqnarray*}

A positive *Z* statistic means an increasing trend and a negative *Z* statistic denotes a decreasing trend. If |*Z*| > 1.96, then the null hypothesis is rejected, i.e., the trend is significant at the 95% confidence level, and vice versa.

#### Path analysis

Path analysis is a multivariate statistical technique, proposed by Wright (1921), for analyzing the causal relationship between variables. It is an extension of regression analysis, and can partition direct and indirect correlations to differentiate between correlation and causation (Wright, 1934; Li et al., 2011a; Li et al., 2011b; Zhang et al., 2014). The causal relationships for the impact of climatic factors on runoff in the Qira River basin were analyzed using a path diagram, from which the direct and indirect impacts of climatic factors on the river runoff could be readily identified.

Assuming there to be a dependent variable *y* and multiple independent variables *x*_1_, *x*_2_, …, *x_n_*, the correlation coefficient between any two independent variables, *x_i_* and *x_j_*, is *r_ij_*. The direct path coefficient from the independent variable *x_i_* to the dependent variable *y* is *P_yi_*, while the indirect path coefficient of *x_i_*, through *x_j_* to *y*, is *r_ij_P_yj_*. The sum of the direct and indirect path coefficients (*r_iy_*) of *x_i_* to *y* means the correlation coefficient between *x_i_* and *y*. The expression is given by:

(5) \begin{eqnarray*} \displaystyle {r}_{i y}={p}_{y i}+\sum _{j=i+1}^{n}{r}_{i j}{p}_{y j}.&&\displaystyle \end{eqnarray*}

**Figure 2:**
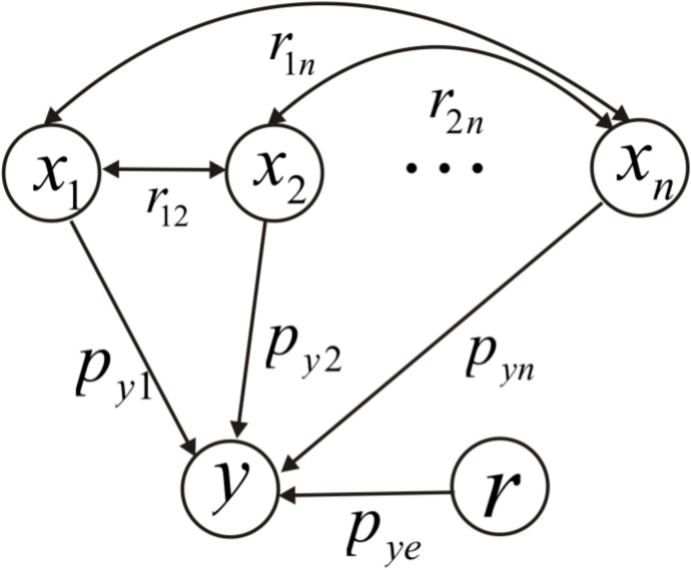
Schematic diagram of path analysis.

Figure 2 shows a schematic diagram of path analysis, describing the independent variables (*x*_1_, *x*_2_, …*x_n_*) and a dependent variable (*y*) with a residual term (*r*). Arrows represent the causal relationships. *x*_1_ → *y*, *x*_2_ → *y*, …, *x_n_* → *y* denote direct paths with direct path coefficients of *P*_*y*1_, *P*_*y*2_, …, *P_yn_*, respectively. *x*_1_↔*x*_2_, …, *x*_*n*−1_↔*x_n_* refer to correlations among variables with correlation coefficients for *r*_12_, …, *r*_*n*−1,*n*_, respectively. *x*_1_↔*x*_2_ → *y*…, *x*_*n*−1_↔*x_n_* → *y* are indirect paths with path coefficients *r*_12_*P*_*y*2_, …, *r*_*n*−1,*n*_*P_yn_*, respectively. *r* → *y* is the path of the residual term with path coefficient *P_ye_*.

#### Mann–Whitney test

The Mann–Whitney test is extensively used to determine abrupt changes in climatic and hydrological time series (Ling et al., 2011). The abrupt-change points in meteorological and hydrological series were explored in this research. Given the variable series *X* = *x*_1_, *x*_2_, …*x_n_*, the time series can be partitioned into two parts: *Y* = *x*_1_, *x*_2_, …*x*_*n*_1__ and *Z* = *x*_*n*_1_+1_, *x*_*n*_1_+2_, …, *x*_*n*_1_+*n*_2__. The Mann–Whitney abrupt-change test statistic is expressed as:

(6) \begin{eqnarray*} \displaystyle U=\frac{\displaystyle \sum _{t=1}^{n 1}R({x}_{t})-\frac{{n}_{1}({n}_{1}+{n}_{2}+1)}{2}}{[{n}_{1}{n}_{2}({n}_{1}+{n}_{2}+1)/12]^{1/2}},&&\displaystyle \end{eqnarray*}

where *R*(*x_t_*) is the rank of the variable series. If |*Z*| > 1.96, then the null hypothesis *H*_0_ is rejected, i.e., abrupt change is significant at the 95% confidence level, and vice versa.

#### Wavelet analysis

Wavelet analysis is a time-frequency analysis method that can characterize local features in time and frequency domains, and has been proven as a powerful technique for insight into the periodicity of climate variables and runoff time series without requiring the time series to be stationary (Torrence & Compo, 1998; Labat, 2005; Xu et al., 2013).

Assuming that $f\left(t\right)$ is a discrete signal, such as the time series of climatic factors or runoff, a continuous wavelet function $\varphi \left(\tau \right)$ is expressed by:

(7) \begin{eqnarray*} \displaystyle \varphi (\tau )=\varphi (a,b)=\vert a\vert ^{-1/2}\varphi \left(\frac{t-b}{a}\right),&&\displaystyle \end{eqnarray*}

where *t* refers to time, and *a* and *b* are the scale and translation parameters, respectively. The continuous wavelet transform of the discrete signal is defined by the convolution of $f\left(t\right)$ and a continuous wavelet function $\varphi \left(\tau \right)$:

(8) \begin{eqnarray*} \displaystyle {W}_{f}(a,b)=\vert a\vert ^{-1/2}\int \nolimits _{-\infty }^{+\infty }f(t){\varphi }^{\ast }\left(\frac{t-b}{a}\right)d t,&&\displaystyle \end{eqnarray*}

where *W_f_*(*a*, *b*) indicates the wavelet coefficient, and ${\varphi }^{\ast }\left(\tau \right)$ is the conjugate function of $\varphi \left(\tau \right)$. The wavelet variance is used to detect the existing periods in the signal. This can be given by:

(9) \begin{eqnarray*} \displaystyle {W}_{f}(a)=\int \nolimits _{-\infty }^{+\infty }\vert {W}_{f}(a,b)\vert ^{2}d b.&&\displaystyle \end{eqnarray*}

The Morlet wavelet function with non-orthogonality was selected in this study as the continuous wavelet function to analyze the characterization of periodicity in climatic factors and runoff time series.

#### Correlation dimension

The correlation dimension approach has been widely used to determine if climatic and hydrological time series show chaotic dynamic characteristics (Sivakumar, 2007; Xu et al., 2010). This is important for providing information on embedding the attractor and determining the number of parameters that exist in the evolution of the variable.

Suppose that $f\left(t\right)$ is the time series of a variable, such as climatic factors or runoff, it is generated via a nonlinear system with *n* degrees of freedom. The series of state vectors [${f}^{(n)}\left(t\right)$] is constructed in the *n*-dimensional phase space:

(10) \begin{eqnarray*} \displaystyle {f}^{(n)}(t)=\left\{f(t),f(t+\tau ),\ldots ,f(t+(n-1))\tau \right\},&&\displaystyle \end{eqnarray*}

where *n* denotes the embedding dimension and *τ* is the time delay. If there are deterministic dynamics in the nonlinear system, the trajectory in the phase space converges towards the “attractor.” The correlation dimension of the attractor is calculated by the G-P method (Grassberger & Procaccia, 1983). For a detailed description of the G-P method, readers are referred to Xu et al. (2010). Briefly, however, the expressions are given as:

(11) \begin{eqnarray*} \displaystyle C(r)=\frac{1}{{N}_{r}^{2}}\sum _{j=1}^{{N}_{r}}\sum _{i=1}^{{N}_{r}}\Theta (r-\vert {f}_{i}(t)-{f}_{j}(t)\vert ),&&\displaystyle \end{eqnarray*}

(12) \begin{eqnarray*} \displaystyle \Theta (x)=\left\{\begin{array}{ll} \displaystyle 0,&\displaystyle x\leq 0\\ \displaystyle 1,&\displaystyle x> 0, \end{array}\right.&&\displaystyle \end{eqnarray*}

(13) \begin{eqnarray*} \displaystyle {N}_{r}=N-(m-1)\tau ,&&\displaystyle \end{eqnarray*}

where *r* and *N_r_* are the surveyor’s rod for distance and the number of reference points from *N*, which is the number of points. When the hypersphere of the radius, *r* → 0, the correlation exponent (*d*), i.e., the correlation dimension of the attractor, is expressed by

(14) \begin{eqnarray*} \displaystyle C(r)\propto {r}^{d},&&\displaystyle \end{eqnarray*}

the correlation exponent (*d*) is the slope coefficient of $\ln \left(C\left(r\right)\right)$ versus *lnr*, and can be solved by the least-squares method. In this study, the chaotic dynamic characteristics of the temperature, precipitation and runoff time series were analyzed using the correlation dimension approach.

#### *R*/*S* analysis

The *R*/*S* analytic method, which is a fractal theory for analyzing long-term correlation characteristics of variable series (Li et al., 2008; Xu et al., 2010), has been widely used in meteorology, hydrology and other fields (Feng et al., 2008). Considering *X*_1_, *X*_2_, …, *X_N_* as the time series of variables, such as annual mean air temperature, accumulated precipitation and runoff, for a period of time *N* it will be characterized by the mean ($\bar {X}$), the standard deviation (*S*) and the range (*R*), which can be briefly calculated via the following expressions:

The mean value series is given as

(15) \begin{eqnarray*} \displaystyle \overline{X}=\sum _{i=1}^{n}({X}_{i})/N.&&\displaystyle \end{eqnarray*}

The accumulative deviation is

(16) \begin{eqnarray*} \displaystyle X(N)=\sum _{i=1}^{N}({X}_{i}-\overline{X}).&&\displaystyle \end{eqnarray*}

The extreme deviation is written by

(17) \begin{eqnarray*} \displaystyle R(N)={m a x}_{1\leq i\leq n}(X(i))-{m i n}_{1\leq i\leq n}(X(i));&&\displaystyle \end{eqnarray*}

The standard deviation is

(18) \begin{eqnarray*} \displaystyle S={\left[\frac{1}{N}\left(\sum _{i=1}^{N}({X}_{i}-\overline{X})^{2}\right)\right]}^{\frac{1}{2}}.&&\displaystyle \end{eqnarray*}

The *R*/*S* is proposed by the empirical law (Hurst, 1951)

(19) \begin{eqnarray*} \displaystyle R/S=(\alpha N)^{H},&&\displaystyle \end{eqnarray*}

where *H* is the so-called Hurst exponent. According to (ln(*N*), ln(*R*/*S*)), *H* is the slope coefficient, which can be obtained by the least-squares method in a log–log grid.

The Hurst exponent (*H*) reflects a long range correlation of a time series and its value ranges between 0 and 1. When *H* > 0.5, the time series has a long-term, enduring characteristic, and the future trend of variable series will be consistent with the past. That is, if the past trend of a series was increasing, the future will also indicate an increasing trend. When *H* = 0.5, the time series is completely independent, and the change is stochastic or only a short-range correlation (Joan et al., 1991). When *H* < 0.5, the time series has a long-range anti-persistence and its future tendency will be the opposite change. That is, if the trend of a series was increasing, the future will show a decreasing trend.

## Results

### Linear characteristics of climatic factors and runoff

#### Variation trends in average temperature, accumulated precipitation and runoff

**Figure 3:**
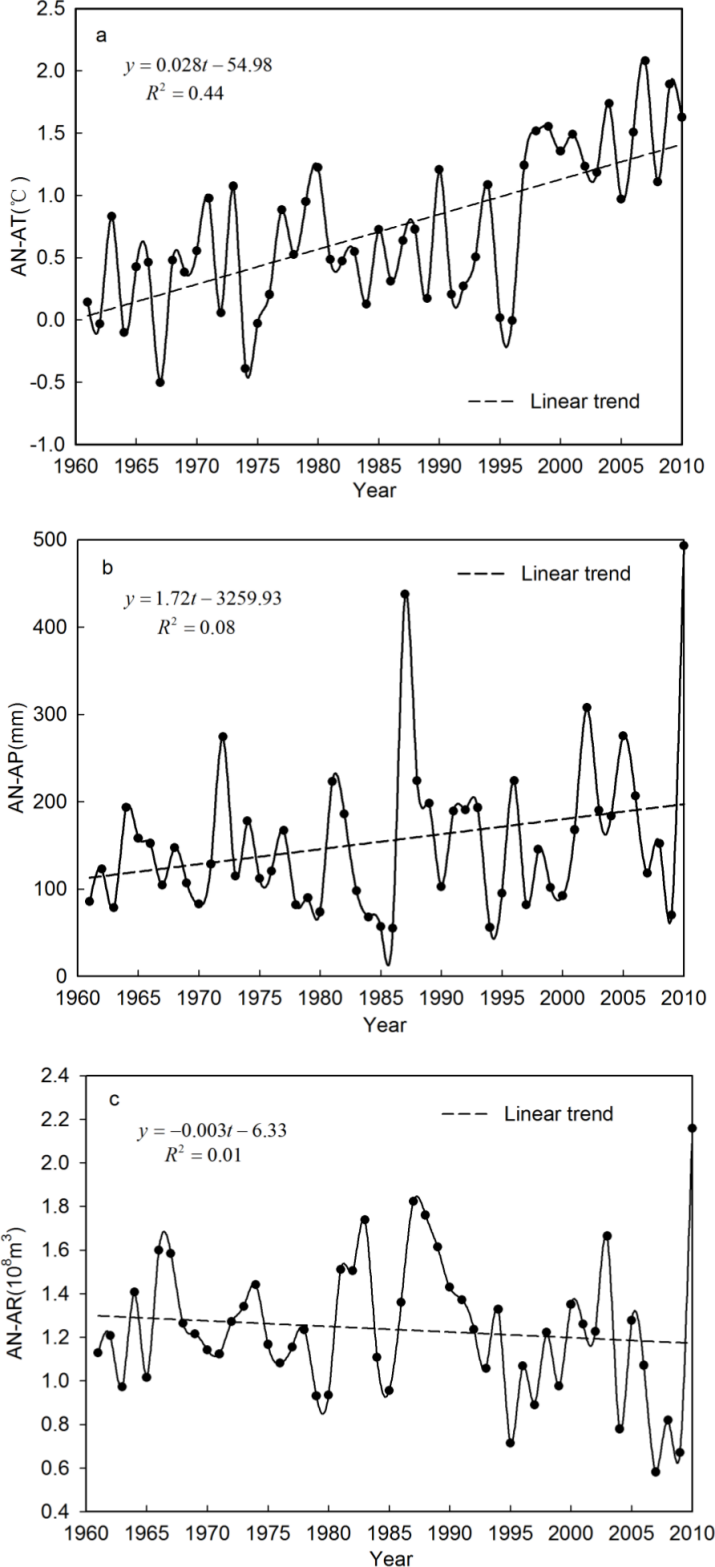
Linear trend of AN-AT, AN-AP and AN-AR time series during the period 1961–2010 in the Qira River basin, respectively.

The linear trends in the annual average temperature (AN-AT), annual accumulated precipitation (AN-AP) and runoff (AN-AR) time series during the study period (1961–2010) in the Qira River basin were investigated using regression analysis (Fig. 3). The time series of AN-AT and AN-AP showed a total increase, with a rate of increase of 0.28 °C/10a and 17.2 mm/10a during the last 50 years, respectively; whereas, the time series of AN-AR indicated a slight decrease, at a rate of −0.03 × 10^8^ m^3^/10a.

The statistical significance of the variation trends in the AN-AT, AN-AP and AN-AR time series was assessed using the Mann–Kendall test. The statistic of the nonparametric Mann–Kendall trend test for the AN-AT time series was *z* = 4.78 > *z*_0.025_ = 1.96, which meant that the null hypothesis was rejected during the study period (1961–2010), i.e., the AN-AT time series in the Qira River basin during that period possessed a significant increasing trend at the 0.05 confidence level. However, the result for the AN-AP time series was *z* = 1.30 < *z*_0.025_ = 1.96, indicating that the increasing trend of the AN-AP time series in the Qira River basin was not significant at the 0.05 confidence level. Similarly, the AN-AR time series result, *z* = − 1.05 > *z*_0.025_ = − 1.96, meant that the null hypothesis could not be rejected for the study period (1961–2010). The decrease in the AN-AR time series in the Qira River basin was not a statistically significant trend at the 0.05 confidence level.

The variation trends of seasonal average temperatures during the period 1961–2010 in the Qira River basin, including that of the spring average temperature (SP-AT), summer average temperature (SU-AT), autumn average temperature (AU-AT) and winter average temperature (WI-AT), were identified using regression analysis and the Mann–Kendall trend test (Table 1). All the seasonal average temperature time series increased significantly at the 0.05 confidence level in the last 50 years. The rates of increase in the SP-AT and AU-AT time series were comparable with that of the AN-AT time series. However, among all the seasonal average temperatures, the rates of increase in the SU-AT and WI-AT time series were the smallest and largest, respectively, implying that the temperature in winter featured the most prominent increase, while the temperature in summer increased more slowly during the past 50 years.

**Table 1:** Variation rate of regression analysis and Mann–Kendall trend test for seasonal temperature, precipitation and runoff in the Qira River basin.

Variable	Period	Variation rate	*Z*	*H*_0_	Trend	Significance^*^
Temperature	SP-AT	0.24 °C/10a	2.56	R	Increase	Significant
	SU-AT	0.10 °C/10a	2.11	R	Increase	Significant
	AU-AT	0.28 °C/10a	3.85	R	Increase	Significant
	WI-AT	0.42 °C/10a	3.63	R	Increase	Significant
Precipitation	SP-AP	0.21 mm/10a	0.00	A	Increase	Insignificant
	SU-AP	9.38 mm/10a	1.46	A	Increase	Insignificant
	AU-AP	0.61 mm/10a	1.81	A	Increase	Insignificant
	WI-AP	1.72 mm/10a	0.11	A	Increase	Insignificant
Runoff	SP-AR	−0.01(10^8^ m^3^/10a)	−1.29	A	Decrease	Insignificant
	SU-AR	−0.03(10^8^ m^3^/10a)	−0.81	A	Decrease	Insignificant
	AU-AR	−0.004(10^8^ m^3^/10a)	−0.62	A	Decrease	Insignificant
	WI-AR	0.002(10^8^ m^3^/10a)	1.09	A	Increase	Insignificant

**Notes.**

R and A stand for rejected and accepted, respectively.

* denotes the significance at 0.05 significance level.

Similarly, the seasonal accumulated precipitation time series during the period 1961–2010 in the Qira River basin, including the spring accumulated precipitation (SP-AP), summer accumulated precipitation (SU-AP), autumn accumulated precipitation (AU-AP) and winter accumulated precipitation (WI-AP) time series, were also explored, using the same methods (Table 1). All of the seasonal accumulated precipitation time series showed insignificant increases at the 0.05 confidence level in the last 50 years. Among them, the rate of increase in SU-AP was the largest, meaning the increase in annual accumulated precipitation was concentrated mainly in summer. Meanwhile, the rate of increase in the WI-AP time series was also greater than that of the others, implying an increase in snowfall in winter during the last 50 years.

The linear trends in the spring accumulated runoff (SP-AR), summer accumulated runoff (SU-AR), autumn accumulated runoff (AU-AR) and winter accumulated runoff (WI-AR) time series during the study period (1961–2010) in the Qira River basin were also analyzed, again using the same methods (Table 1). The SP-AR, SU-AR and AU-AR time series showed an insignificant decreasing trend at the 0.05 confidence level during the last 50 years. Only the WI-AR time series showed an insignificant increasing trend at the 0.05 confidence level, with a rate of 0.002 × 10^8^ m^3^/10a. This is because the increase in snowfall, as well as the increment of glacier-/snowmelt with the rise in temperature in winter, would have led to a simultaneous increase in runoff.

#### Correlation analysis between average temperature, accumulated precipitation and accumulated runoff

According to the criterion of multiple linear regression analysis, there is a significant linear correlation relationship between every independent variable and the dependent variable. Therefore, path analysis, as a special paradigm of multiple regression analysis, is necessary to verify the linear significance between the independent variables and dependent variable. Moreover, before using path analysis to reveal the impact of the independent variables on the dependent variable, which is required to follow the normal distribution, the normality of the dependent variable needs be tested via the Kolmogorov–Smirnov test (Frank & Massey, 1951).

Correlation analysis was used to verify the linear significance between the temperature, precipitation and runoff. Table 2 shows the Pearson correlation coefficients between the temperature, precipitation and runoff at the annual and seasonal level for the Qira River basin. The correlation coefficient between AN-AT and AN-AR, and between AN-AP and AN-AR, was significant (values of −0.248 and 0.545 at the 0.01 confidence level, respectively). This implies that the closest linear relationship in the Qira River basin existed between runoff and precipitation, while temperature showed a negative effect on runoff due to strong evaporation and/or infiltration.

**Table 2:** Pearson correlation coefficients between temperature, precipitation and runoff based on annual and seasonal level in the Qira River.

Vaviable	AN-AR	SP-AR	SU-AR	AU-AR	WI-AR
AN-AT	−0.248^**^				
SP-AT		−0.281			
SU-AT			−0.337^**^		
AU-AT				−0.097	
WI-AT					0.335^*^
AN-AP	0.545^**^				
SP-AP		0.291^*^			
SU-AP			0.403^**^		
AU-AP				0.087	
WI-AP					−0.046

**Notes.**

* signifies that correlation is significant at 0.05 level.

** signifies that correlation is significant at 0.01 level.

The correlation between SU-AT and SU-AR at the seasonal level, as well as between SU-AP and SU-AR, was significant (*P* < 0.01); whereas, between WI-AT and WI-AR, and between SP-AP and SP-AR, the correlation was significant, at the 0.05 confidence level. The other variables in the four seasons showed insignificant correlation at the *α* ≤ 0.05 significance level. The correlation coefficients between SP-AT, SU-AT, AU-AT and the corresponding seasonal runoff were negative values, implying that strong evaporation and/or infiltration weakened the increase in runoff as temperatures increased. However, the correlation coefficient between WI-AT and WI-AR was 0.335, which was larger than that between WI-AP and WI-AR (−0.046). Precipitation in the form of snowfall, in winter, is accumulated as the snowpack. The process of glacier-/snowmelt in the mountains then begins and directly contributes to runoff when the temperature increases significantly in winter. This is why only the contribution of temperature showed a significant and positive correlation with runoff.

In addition, the correlation coefficient between SP-AP and SP-AR, and that between SU-AP and SU-AR, was also significant and positive (0.291 and 0.403, respectively). This reveals that some of the precipitation in spring was able to occur as rainfall, which may have contributed directly to runoff, and the precipitation in summer was the dominant recharge resource for runoff. These results indicate that the correlation between the precipitation and runoff in spring, summer and autumn was closer than that between temperature and runoff, while the opposite was true in winter.

#### Impacts of temperature and precipitation on runoff

The requirement for path analysis is that there is a significant linear correlation between independent and dependent variables. Meanwhile, the dependent variables need to follow the normal distribution. In the present study, these two conditions were only satisfied at the annual and summer level, in which the linear correlations between temperature, precipitation and runoff were significant (*P* < 0.01), and accumulated runoff was found to follow a normal distribution based on the Kolmogorov–Smirnov statistics of 0.087 (*P* < 0.05) and 0.089 (*P* < 0.05), respectively. Therefore, path analysis was implemented at the annual and summer level to investigate the impacts of temperature and precipitation on runoff in the Qira River basin (Table 3).

**Table 3:** Path coefficients of direct and indirect effects of AT and AP on runoff in the Qira River basin.

Variable	Direct effect	Indirect effect		Total
		AT	AP	
AN-AT	−0.280^*^		0.032	−0.296
AN-AP	0.561^*^	−0.016		0.593
SU-AT	−0.366^*^		0.151	−0.497
SU-AP	0.423^*^	−0.131		0.574

**Notes.**

* signifies that path coefficient is significant at the 0.01 level.

The direct effect of AN-AT on AN-AR was −0.280, and the indirect effect of AN-AP was 0.032l within a year. The total effect of AN-AT was −0.296. The direct effect of AN-AP on AN-AR was 0.561, and the indirect effect of AN-AT via AN-AP on AN-AR was −0.016 during a year. The total effect of AN-AP within a year reached 0.593. The results via path analysis reveal that the impact of precipitation on runoff formation constituted the main driving effect, while that of temperature on runoff showed a negative effect, implying that the occurrence of strong evaporation and/or infiltration, as temperatures increased, indirectly weakened the increase in runoff in the Qira River basin. In addition, the path coefficient of the residual term of AN-AR was 0.791 (*P* < 0.01), implying that AN-AR was also influenced by other causal factors. Additional factors (e.g., land cover, soil moisture) should therefore be considered in future studies.

Similarly, in summer, the direct effects of SU-AT and SU-AP on AN-AR were −0.366 and 0.423, respectively, and their indirect effects were 0.151 and −0.131, respectively. The total effect of SU-AT and SU-AP on runoff was −0.497 and 0.574, respectively. This also means that, in summer, the main impact upon runoff was from precipitation and, annually, precipitation was the main contributor to runoff. However, the temperature in summer showed a weakening effect on the runoff. Moreover, the path coefficient of the residual term of SU-AR was 0.839 (*P* < 0.01), implying that other factors also influenced SU-AR. These factors should be investigated in further work.

### Nonlinear characteristics of climatic factors and runoff

#### Abrupt changes in average temperature, accumulated precipitation and runoff

**Figure 4:**
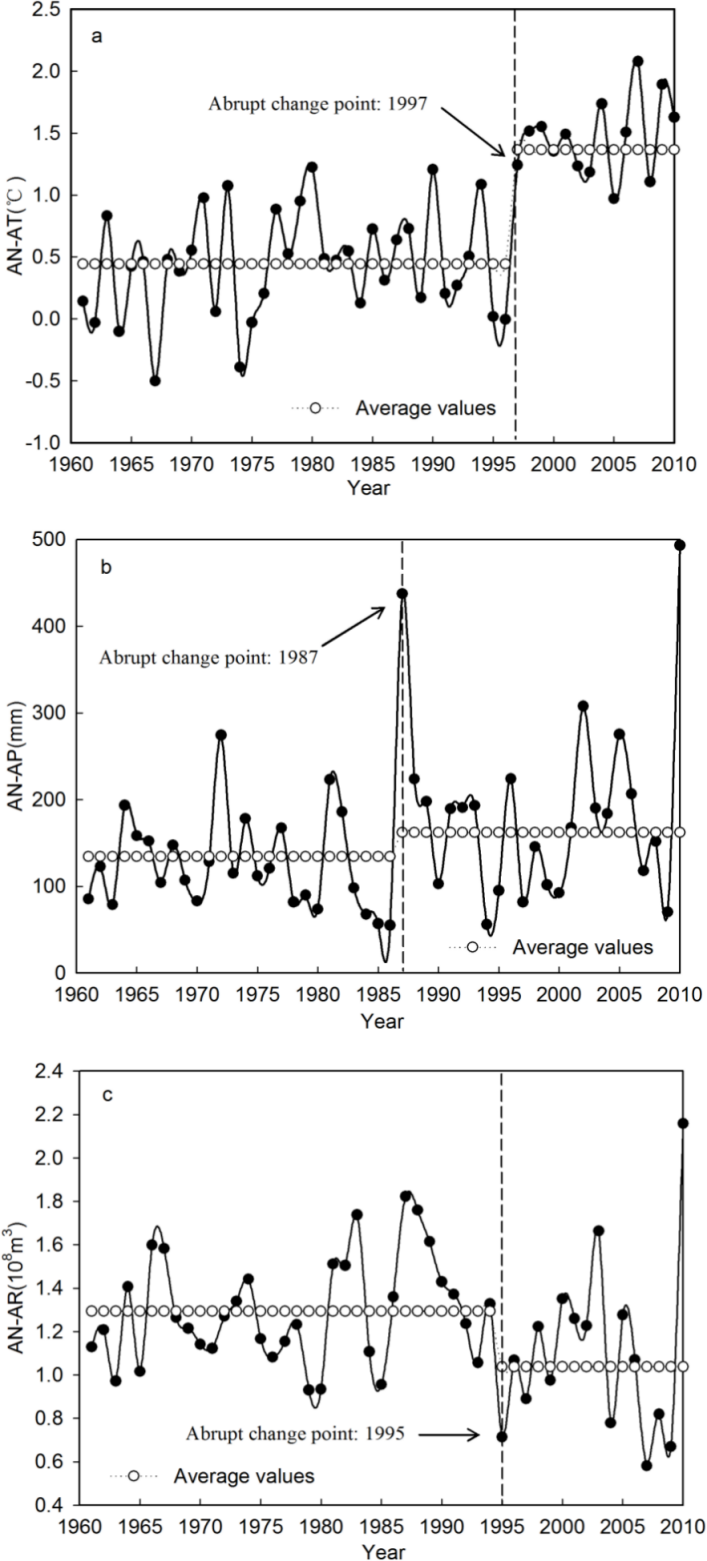
Abrupt change of the AN-AT, AN-AP and AN-AR during the period 1961–2010 in the Qira River basin.

Hydro-climatic processes constitute a highly complicated nonlinear system, and meteorological and hydrological time series generally show characteristics of fluctuation and nonlinear change. Abrupt changes tend to exist in the time series of variables due to changes in the global climate and the influence of human activities. Analysis of the AN-AT, AN-AP and AN-AR time series, for the period 1961–2010 in the Qira River basin, indicated that abrupt changes occurred in 1997, 1987 and 1995, respectively (Fig. 4). The abrupt changes in these time series were verified by applying the Mann–Whitney test. The results showed that the null hypothesis (*H*_0_) was rejected at the 0.05 confidence level, i.e., abrupt changes did indeed occur, at a statistically significantly level, around 1997, 1987 and 1995 for AN-AT, AN-AP and AN-AR, in the Qira River basin, respectively (Table 4). The mean values of AN-AT, AN-AP and AN-AR before the abrupt changes were 0.44 °C, 134.48 mm and 1.29 × 10^8^ m^3^, respectively, and, after the changes, were 1.37 °C, 162.02 mm and 1.04 × 10^8^ m^3^ respectively. The increment rates for AN-AT and AN-AP were 207.40% and 20.48%, respectively, while for AN-AR it was −19.81%, after the abrupt changes.

**Table 4:** Mann–Whitney abrupt change test of the temperature, precipitation and runoff in annual and seasonal time scale during the study period 1961–2010 for the Qira River basin.

Variable	Period	N	U	*H*_0_	ACP	Mean value	RI
AN-AT	1961–1996	36	−5.24^*^	R	1997	0.44	207.40%
	1997–2010	14				1.37	
AN-AP	1961–1986	26	−2.36^*^	R	1987	134.48	20.48%
	1987–2010	24				162.02	
AN-AR	1961–1994	34	2.01^*^	R	1995	1.29	-19.81%
	1995–2010	16				1.04	
SP-AT	1961–1996	36	−4.26^*^	R	1997	1.87	64.90%
	1997–2010	14				3.09	
SU-AT	1961–1996	36	−2.65^*^	R	1997	10.87	4.37%
	1997–2010	14				11.34	
AU-AT	1961–1996	36	−4.63^*^	R	1997	0.61	176.93%
	1997–2010	14				1.68	
WI-AT	1961–1996	36	−3.19^*^	R	1997	−11.41	10.66%
	1997–2010	14				−10.19	
SU-AP	1961–1986	26	−2.24^*^	R	1987	68.21	61.68%
	1987–2010	24				110.29	
SU-AR	1961–1994	34	2.12^*^	R	1995	0.94	−15.81%
	1995–2010	16				0.79	

**Notes.**

R denotes rejected.

ACP and RI stand for abrupt change point and rate of increment, respectively.

* signifies that statistic of Mann–Whitney is significant at the 0.01 level.

Abrupt changes in temperature, precipitation and runoff at the seasonal level in the Qira River basin were also detected, again by using the Mann–Whitney abrupt change test (Table 4). A common point of abrupt change was verified to have occurred in 1997 with regard to average temperature, in all four seasons. This means that the abrupt change of average temperature in each season remained consistent with that of annual average temperature. The average temperature in the four seasons, to differing degrees, increased after the abrupt change. The increment rate for SP-AT, SU-AT, AU-AT and WI-AT was 64.90% , 4.37%, 176.93% and 10.66%, respectively. Abrupt but statistically insignificant changes (at the 0.05 confidence level) in precipitation and runoff in all seasons except summer were detected. The SU-AP and SU-AR time series featured abrupt changes in 1987 and 1995, respectively, implying that the abrupt changes in annual precipitation and runoff were synchronous with those in summer.

#### Multiple time scale analysis of average temperature, accumulated precipitation and runoff

**Figure 5:**
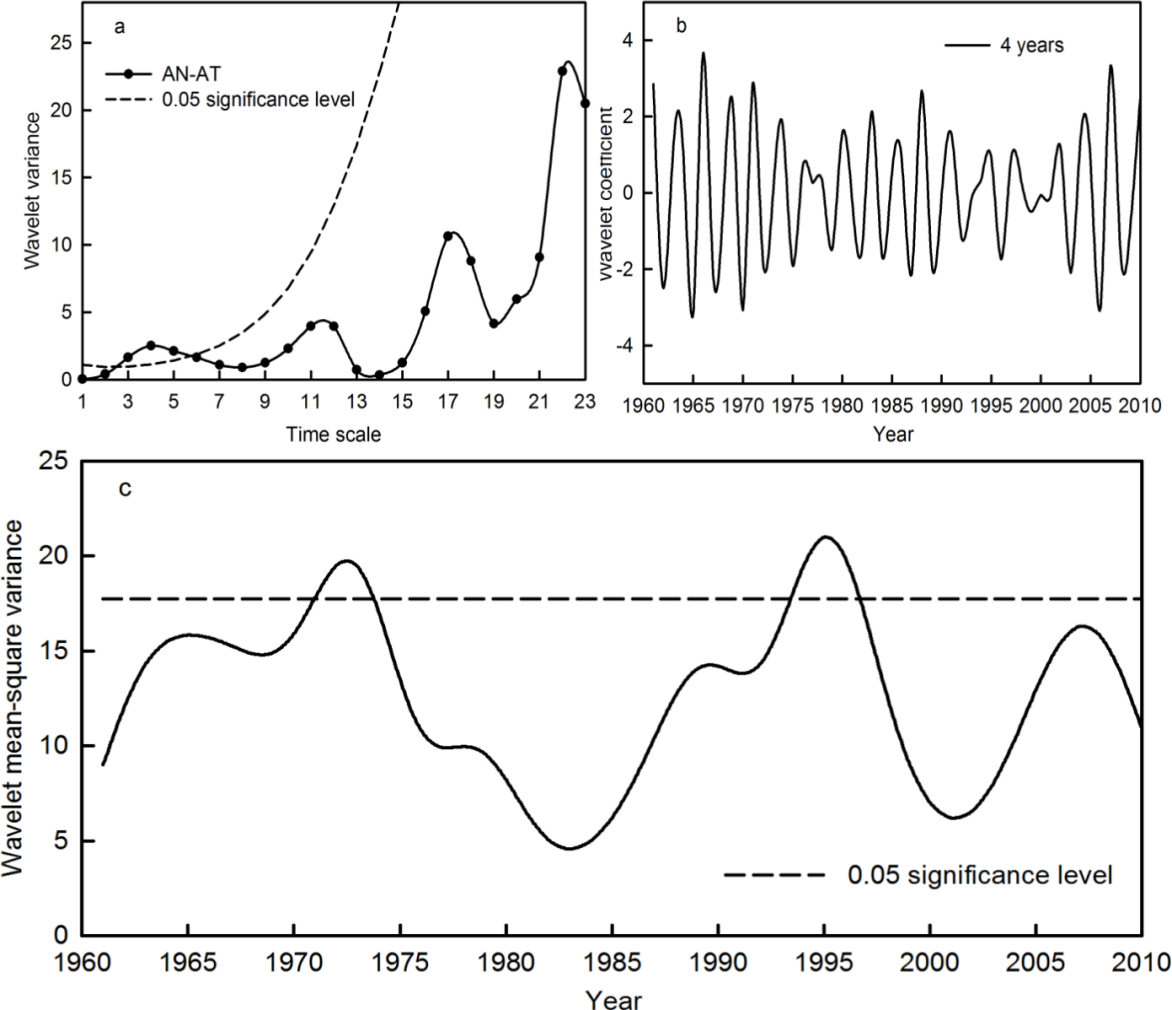
Wavelet variance (A), wavelet coefficient (B) and wavelet mean-square variance (C) of the AN-AT time series during the period 1961–2010 in the Qira River basin.

To further identify the effects of climatic factors at multiple time scales on the runoff in the Qira River basin, wavelet analysis was used to determine the periodicity, phase change and periodic intensity of temperature, precipitation and runoff at these different time scales in the region. The peak values of the wavelet variance of AN-AT occurred in the fourth year, based on the chi-squared test at the 0.05 confidence level (Fig. 5A). This indicates that the AN-AT time series had a significant 4-year period. Moreover, according to the interdecadal variations, the wavelet variance of AN-AT still featured local maximums in the 11th, 17th and 22nd years. This means that the AN-AT time series also possessed periods of 11, 17 and 22 years, albeit not statistically significant at the 0.05 confidence level. On the basis of the wavelet mean-square variance, the most significant periodic fluctuations occurred during 1971–1973 and 1993–1996, at the 0.05 confidence level (Fig. 5C). Meanwhile, the wavelet coefficients of the 4-year period were in a positive phase during 1971–1972 and 1993–1995, when the AN-AT time series showed its high temperature phase. The other years indicated a lower temperature phase (Fig. 5B).

**Figure 6:**
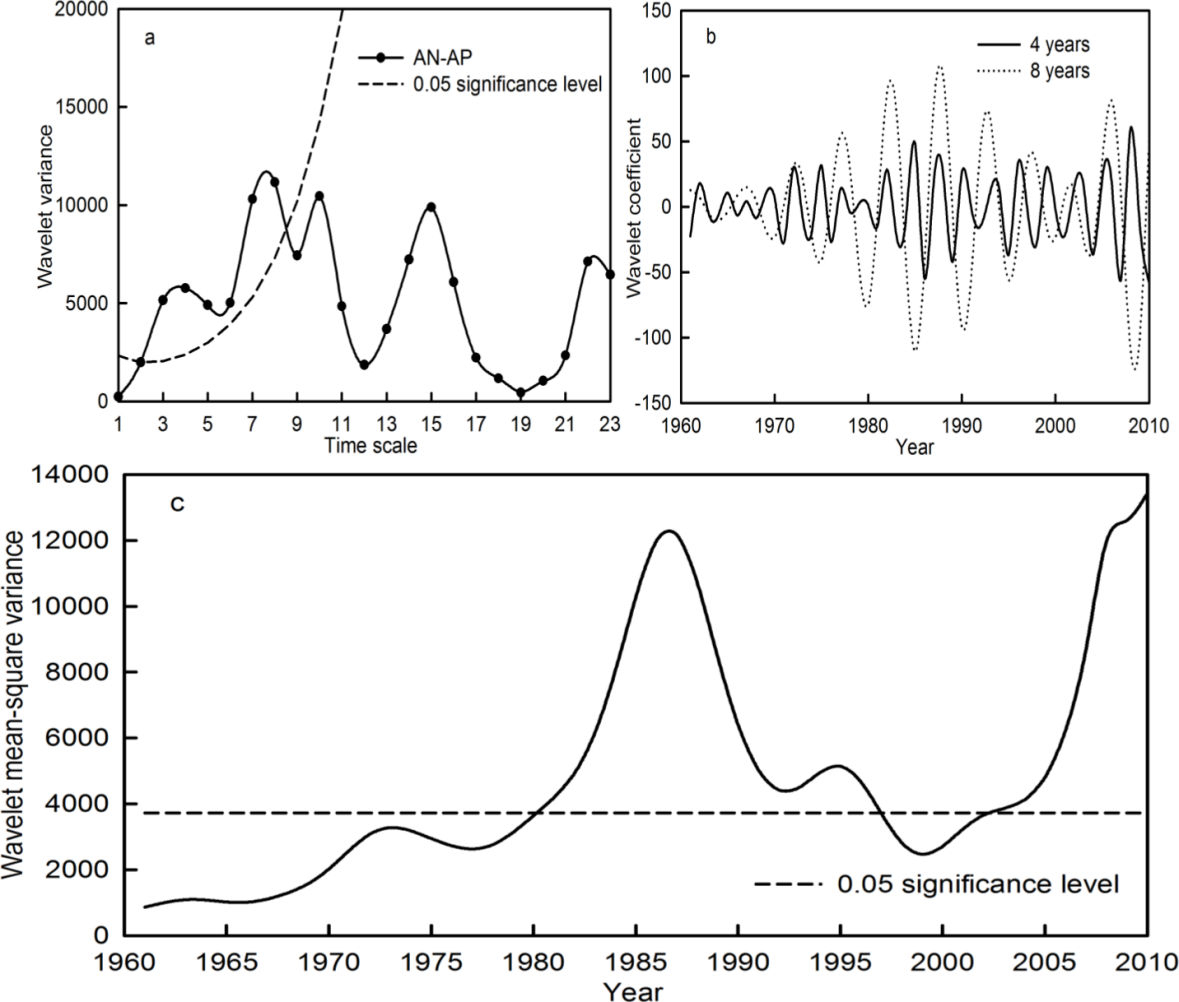
Wavelet variance (A), wavelet coefficient (B) and wavelet mean-square variance (C) of the AN-AP time series during the period 1961–2010 in the Qira River basin.

Similarly, the AN-AP time series in the Qira River basin featured significant periods of 4 and 8 years (Fig. 6A). The periodic changes on the 4- and 8-year time scale, within which the most significant periodic fluctuations occurred during 1980–1997 and 2003–2010, are statistically significant at the 0.05 level (Fig. 6C). The wavelet coefficients revealed alternately high and low precipitation during the significant periodic fluctuations (Fig. 6B). In addition, the AN-AP time series still possessed periods of 10, 15 and 22 years. However, these were not statistically significant at the 0.05 confidence level.

**Figure 7:**
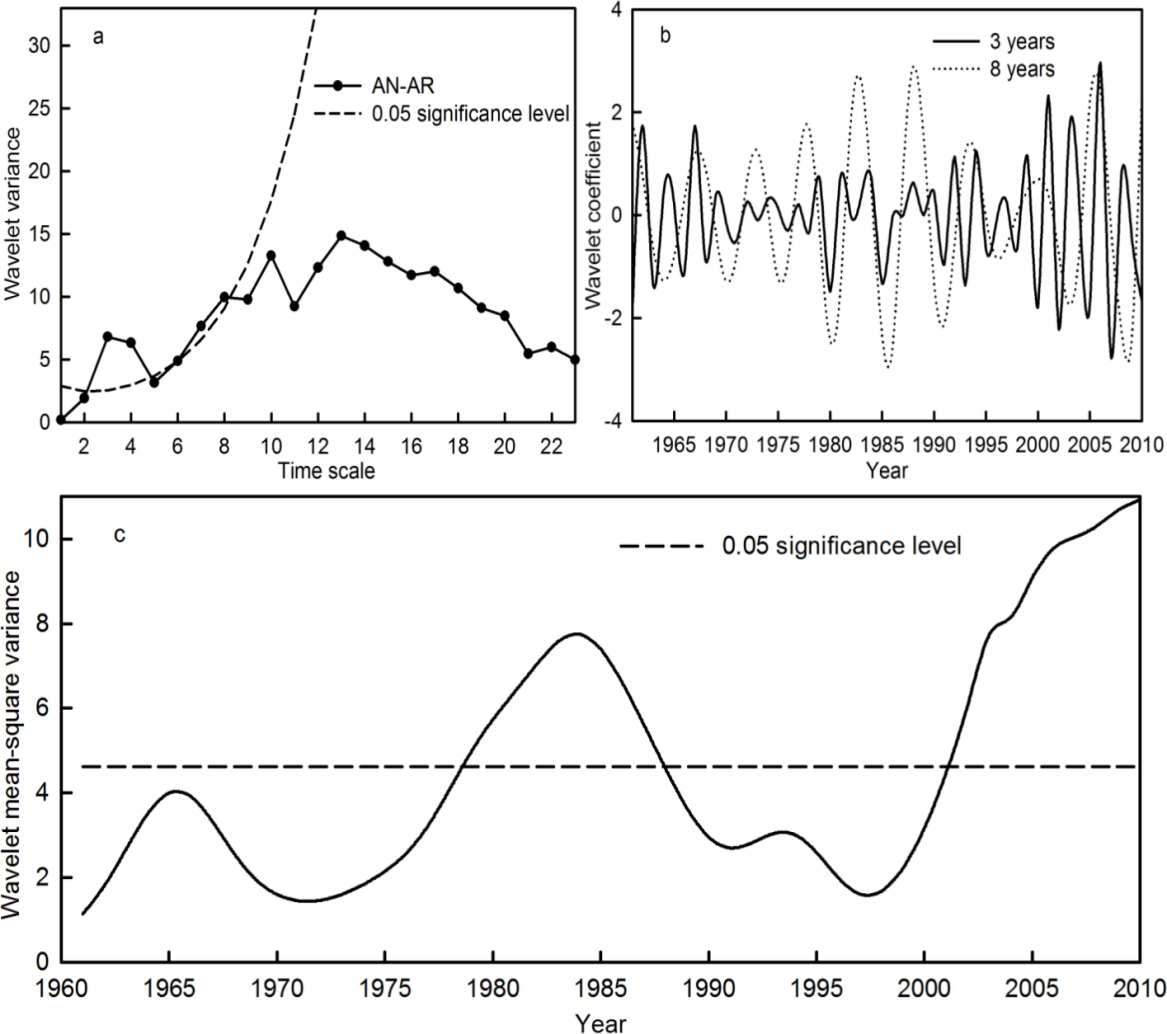
Wavelet variance (A), wavelet coefficient (B) and wavelet mean-square variance (C) of the AN-AR time series during the period 1961–2010 in the Qira River basin.

Figure 7 shows that the AN-AR time series in the Qira River basin possessed significant periods of 3 and 8 years (Fig. 7A). Moreover, statistically insignificant periods of 10, 13 and 22 years still existed within the AN-AR time series. In terms of significant periods, there were remarkable periodic changes in AN-AR during 1978–1988 and 2002–2010 (Fig. 7C). From the wavelet coefficients, the AN-AR time series indicated an alternately high and low flow during the significant periodic fluctuations (Fig. 7B). This implies that the period change of the AN-AR time series was caused by the synthetic effect of the AN-AT and AN-AP series at multiple time scales.

In addition, the periodicity and periodic intensity of average temperature, accumulated precipitation and runoff at the seasonal level in the Qira River basin were explored (Table 5). The significant periods of all the variables at the seasonal level occurred during 3–8 years. The significant periods and intensity of the AU-AT and AU-AR time series were consistent with those of AN-AT and AN-AR, respectively, while the significant period and intensity of SP-AP were basically synchronous with AN-AP in the last 50 years. This implies that the multiple time scale characteristics of annual temperature and runoff in the Qira River basin were consistent with the temperature and runoff in autumn, respectively, while that of annual precipitation was consistent with the precipitation in spring.

**Table 5:** Period and periodic intensity of average temperature, accumulated precipitation and runoff in the seasonal level for the Qira River basin.

**Index**	**SP-AT**	**SU-AT**	**AU-AT**	**WI-AT**
P	5^*^;11;18	3^*^;10;17	4^*^;17;22	5^*^;12;22
SPF	1977–1983 1996–2006	1970–1999	1970–1975 1992–1995	1970–1982 2005–2010
**Index**	**SP-AP**	**SU-AP**	**AU-AP**	**WI-AP**
P	4^*^;8^*^;10;15	4^*^;7^*^;10;15	4^*^;8^*^;13;18	3^*^;14;18
SPF	1982–1996 2002–2010	1977–2010	1981–1996	1976–1980 1998–2010
**Index**	**SP-AR**	**SU-AR**	**AU-AR**	**WI-AR**
P	3^*^;8^*^;16	3^*^;14;22	3^*^;8^*^;12	8^*^;15
SPF	1961–1991	1977–1988 2000–2010	1977–1988 2003–2010	1987–1988

**Notes.**

P stands for period, SPF denotes significant periodic fluctuation.

* signifies that period is significant at the 0.05 level.

#### Chaotic dynamic characteristics of average temperature, accumulated precipitation and runoff

**Figure 8:**
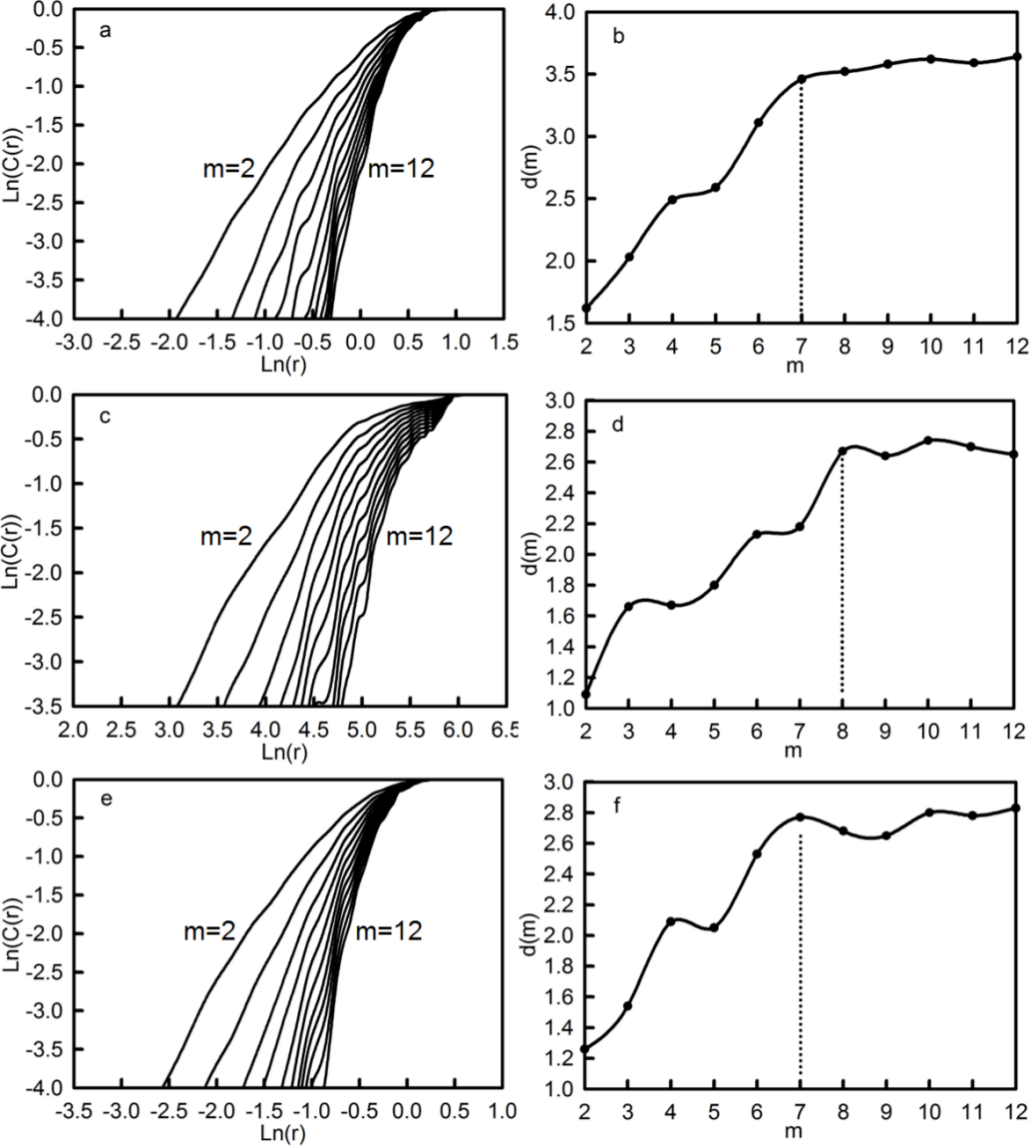
Relationship between ln(*C*(*r*)) and ln(*r*) in different m values for the AN-AT (A), AN-AP (C), AN-AR (E), and that between the correlation exponent (*d*(*m*)) and embedding dimension (*m*).

The correlation dimension method is appropriate for identifying chaotic dynamic characteristics of variables. The AN-AT, AN-AP and AN-AR time series in the Qira River basin were reconstructed in phase space, and the values of the correlation integrals, *C*(*r*), in the different values of the radius, *r*, were calculated. The relationship between ln(*C*(*r*)) and ln(*r*) for AN-AT, AN-AP and AN-AR, with different embedding dimensions, *m*, are shown in Figs. 8A, 8C and 8E, respectively. The correlation exponent (*d*(*m*)), which denotes the slope coefficient of ln(*C*(*r*)) versus ln(*r*), increased with embedding dimension and gradually reached a saturated correlation exponent, i.e., the correlation dimension of attractor (*d_s_*).

Figures 8B, 8D and 8F demonstrate the relationship between the correlation exponent (*d*(*m*)) and embedding dimensions (*m*) of AN-AT, AN-AP and AN-AR, respectively. When the embedding dimensions (*m*) were, respectively, 7, 8 and 7, and the corresponding correlation dimension of attractor (*d_s_*) was 3.46, 2.67 and 2.77, the correlation dimension reached saturation and trended towards stabilization. Because all the values of *d_s_* were greater than 2, and were not equal to integers, this revealed that the AN-AT, AN-AP and AN-AR time series in the Qira River basin featured fractal characteristics, representing complex chaotic systems with a limited degree of freedom. If the dynamic system of variables was expressed, at least the nonlinear differential equations of 4, 3 and 3 independent variables and, at most, those of 7, 8 and 7 independent variables, were needed to describe the dynamic change characteristics of the AN-AT, AN-AP and AN-AR time series, respectively.

Moreover, the embedding dimensions (*m_s_*) and attractor dimensions (*d_s_*) of average temperature, accumulated precipitation and runoff at the seasonal level are shown in Table 6. All the seasonal temperature, precipitation and runoff results remained consistent with their respective annual results, except for the embedding dimension (*m_s_*) and attractor dimension (*d_s_*) of the average temperature in autumn and accumulated precipitation in spring. They required at least the nonlinear differential equations of 3 and 4 independent variables and, at most, those of 6 and 7 independent variables, to describe the dynamic change characteristics of the AU-AT and SP-AP time series, respectively.

**Table 6:** Embedding dimension (*m_s_*) and attractor dimension (*d_s_*) for average temperature, accumulated precipitation and runoff in the seasonal level for the Qira River basin.

Index	AT				AP				AR			
	SP	SU	AU	WI	SP	SU	AU	WI	SP	SU	AU	WI
*m_s_*	7	7	6	7	7	8	8	8	7	7	7	7
*d_s_*	3.45	3.41	2.25	3.43	3.87	2.81	2.72	2.38	2.76	2.62	2.94	2.83

**Notes.**

*m_s_* and *d_s_* denote embedding dimension and attractor dimension, respectively.

#### Long-term memory characteristics of average temperature, accumulated precipitation and runoff

**Figure 9:**
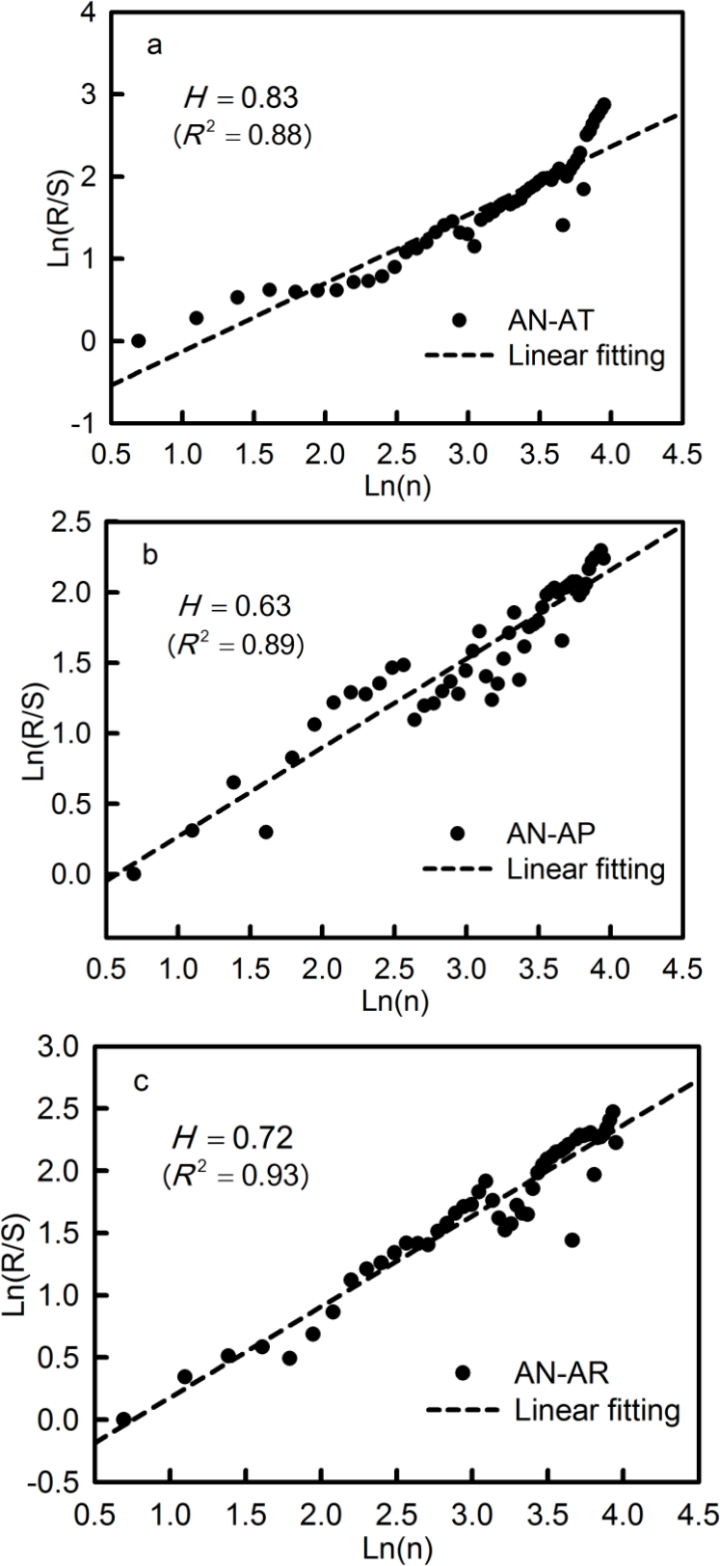
*R*/*S* analysis of the AN-AT (A), AN-AP (B), and AN-AR (C) in the Qira River basin.

*R*/*S* analysis is able to identify the fractal of variables to reveal the long-term memory very well. To determine the continuity of change tendency of both climatic factors and runoff in the future, Fig. 9 shows scatter diagrams and linear fitting curves of the *R*/*S* analyses of the AN-AT, AN-AP and AN-AR time series, based on the abscissa axis for the logarithm of lag time *n* and the ordinate axis for the logarithm of *R*/*S* values, respectively. The values of *H* for the AN-AT, AN-AP and AN-AR time series were greater than 0.5. This means that they were predicted to continue to persist into the future. The *H* value of AN-AR, being between the *H* values of AN-AT and AN-AP, implies that the runoff will be persistently affected by both temperature and precipitation in the future.

Since AN-AT and AN-AP increased gradually over the last 50 years, they will therefore likely continue to do so in the future. AN-AR decreased slightly during the period 1961–2010, implying that annual runoff in the Qira River basin will go on decreasing in the future.

The continuity of change tendency of average temperature, accumulated precipitation and runoff at the seasonal level was investigated based on the results presented in Table 7. All the seasonal variables showed long-term persistent memory, except runoff in autumn, which showed anti-persistency in the future. This implies that the seasonal temperature, precipitation and runoff will maintain their change tendencies in the future, except that runoff in autumn will show a reversed change and gradually increase in time.

**Table 7:** Hurst exponents and R-squared for average temperature, accumulated precipitation and runoff in the seasonal level for the Qira River basin.

Index	AT				AP				AR			
	SP	SU	AU	WI	SP	SU	AU	WI	SP	SU	AU	WI
*H*	0.76	0.65	0.73	0.83	0.68	0.63	0.58	0.69	0.61	0.73	0.44	0.85
*R* ^2^	0.92	0.91	0.89	0.92	0.87	0.90	0.87	0.94	0.92	0.93	0.91	0.87

## Discussion

Hydro-climatic processes represent a highly complicated system featuring both linear and nonlinear characteristics (Jury & Melice, 2000; Ling et al., 2011; Xu et al., 2013). According to previous studies in the Qira River basin, both the significant increase in temperature and insignificant increase in precipitation, at the annual and seasonal levels, are a regional reflection of the climate change from a warming–drying trend to a warming-wetting trend (Shi et al., 2007), while runoff at the annual and seasonal scales is decreasing insignificantly. A possible explanation for this is that the decrease in runoff has resulted from stronger infiltration due to thaw, and increased evapotranspiration due to high temperatures.

In the present study, the average temperature at the annual and seasonal levels showed negative correlation with corresponding runoff, except in winter when there was positive correlation. In addition, it was found, using path analysis, that temperature acted as a negative driving effect on runoff. The main reason for this was probably the low glacial coverage (glacial area of approximately less than 2.68%) in the Qira River basin (Wu, Yang & Hua, 2011). The increase in temperature would result in increased evapotranspiration and, alongside the strong infiltration from thaw in the basin, a gradual reduction in runoff would occur. The change tendency of the runoff would still be a decreasing one even if the proportion of glacial water feeding the runoff increased. This is because smaller glaciers are more sensitive to climatic warming than larger ones (Ye, Ding & Liu, 2001). There is little doubt that temperature in winter has shown the most significant increases in Northeast China (Liu et al., 2003; Qian & Lin, 2004; Jiang, Wang & Lang, 2004; Zhao et al., 2013). The precipitation in winter, as snowfall, is temporarily stored as snowpack and/or ice cover in large alpine regions. Glacier- and snowmelt water is the main recharge source for runoff in winter. Therefore, in winter, temperature and runoff are positively correlated.

The precipitation and runoff during the study period, at the annual and seasonal levels, showed positive correlations, and their correlations were significant annually and in summer. Meanwhile, precipitation was determined, via path analysis, as exerting positive driving effects on runoff. It can be concluded that, although the Qira River is mainly supplied by glacial meltwater and precipitation in the alpine area, precipitation is the major contributor to runoff of the river, especially at the annual and summer-season scales. One good case exemplifying this notion is that the heaviest flood year (in 2010) that the Qira River encountered during the past 50 years was synchronous with the heaviest precipitation year. Since precipitation in alpine regions is stored mainly in solid form as snowpack, which thaws over a long period, runoff is supplied in the following year or more in the presence of increased temperatures. Therefore, abrupt changes in precipitation tend to occur earlier than those of runoff (Ling, Xu & Fu, 2013).

Based on the nonlinear results, it seems that the meteorological and hydrological processes in the Qira River basin possess time-scale dependency, chaotic dynamics, and long-term memory. According to multiple time scale analysis, periodicities and intensities of the temperature and precipitation show mutual supplement or counteraction at different time scales, which determines the main periodicity and intensity of runoff in the Qira River basin. From the correlation dimension of temperature, precipitation and runoff, it was found that the hydro-climatic processes constitute a chaotic, dynamic system, possessing fractal characteristics. This also suggests that complex processes must be introduced into many variables in models in order to better describe the dynamics. On the basis of the Hurst exponent, the temperature, precipitation and runoff were found to possess long-term memories, the main implication of which is that the trends in these variables in the Qira River basin will, in the future, continue their association with climate change patterns that have been apparent in the past.

This study, from a new perspective, has attempted to explore the linearity and nonlinearity of the runoff response to regional climate factors in the Qira River basin. However, due to the particular geographical environment and climate conditions, there was only one long-term meteorological dataset for the basin available for use, and this means there is considerable uncertainty surrounding the results. Therefore, further work should be carried out that uses remote sensing or satellite data (products), coupled with ground-based observations, to improve the availability of meteorological data in the ungauged Qira River basin. Meanwhile, to gain a more detailed understanding of the hydro-climatic processes, further research based on better methods, to complement the findings presented in the present paper, is needed.

## Conclusions

Runoff generation is a quite complicated meteorological and hydrological process affected by many climatic factors (e.g., temperature, precipitation). Based on observed data (including monthly average temperature, accumulated precipitation and runoff), the linear and nonlinear characteristics of the runoff response to temperature and precipitation are revealed during the last 50 years in the Qira River basin. The key conclusions can be summarized as follows: (1) the average temperature at the annual and seasonal level increased significantly during 1961–2010, whereas accumulated precipitation and runoff at the annual and seasonal scales decreased insignificantly, except in winter when it has slightly increased since the 1960s. (2) A significant and positive driving effect, annually and in summer, of precipitation on runoff was found during the study period (1961–2010) by path analysis. However, the impact of temperature on runoff at the same scales was negative, which we attribute to stronger infiltration from thaw and higher evaporation in summer in this extremely arid zone. (3) Abrupt changes in annual average temperature, accumulated precipitation and runoff occurred in 1997, 1987 and 1995, respectively, during 1961–2010. Moreover, their increment rates were, respectively, 207.40% , 20.48%, and −19.81% , after the abrupt changes. (4) The annual average temperature, annual accumulated precipitation and runoff were found to possess 4-year, 4- and 8-year, and 3- and 8-year significant periods, respectively. The periods and intensities between AU-AT and AN-AT, between AU-AR and AN-AR, and between SP-AP and AN-AP were revealed consistently over the last 50 years. (5) The temperature, precipitation and runoff at the annual and seasonal scales showed chaotic, dynamic characteristics, based on the fact—using the correlation dimension analysis method—that none of the correlation dimensions was an integer. These results imply that, in models, complex hydro-climatic processes must be introduced into other variables, in order to better describe the dynamics. (6) It was found that runoff, at the annual and seasonal level, will decrease in the Qira River basin in the future, except in autumn, when it will gradually increase, under the consistent increasing trends in temperature and precipitation.

## Supplemental Information

10.7717/peerj.1104/supp-1 Supplemental Information 1 Runoff, temperature, precipitation
