# Supplementary material for: Linear and nonlinear characteristics of the runoff response to regional climate factors in the Qira River basin, Xinjiang, Northwest China
Source: PeerJ. 2015 Jul 21;3:e1104. doi: 10.7717/peerj.1104 (PMC4517962; doi:10.7717/peerj.1104)
Supplement: Supplemental Information 1 [file peerj-03-1104-s001.pdf]

Time	Annual mean temperature	Annual precipitation	Annual runoff
1961	0.14	85.51	1.13
1962	-0.03	122.71	1.21
1963	0.83	78.62	0.97
1964	-0.10	193.41	1.41
1965	0.43	158.31	1.02
1966	0.46	152.16	1.60
1967	-0.50	104.25	1.58
1968	0.48	147.40	1.26
1969	0.38	106.83	1.21
1970	0.55	82.93	1.14
1971	0.98	128.53	1.12
1972	0.06	274.31	1.27
1973	1.07	114.95	1.34
1974	-0.39	178.00	1.44
1975	-0.03	111.96	1.17
1976	0.20	120.64	1.08
1977	0.88	167.14	1.15
1978	0.52	81.74	1.23
1979	0.95	89.76	0.93
1980	1.22	73.53	0.93
1981	0.49	222.84	1.51
1982	0.47	185.97	1.50
1983	0.55	97.99	1.74
1984	0.13	67.80	1.11
1985	0.73	56.93	0.96
1986	0.31	55.13	1.36
1987	0.64	437.42	1.82
1988	0.73	223.71	1.76
1989	0.17	197.87	1.61
1990	1.21	102.63	1.43
1991	0.21	189.32	1.37
1992	0.27	190.75	1.24
1993	0.51	193.16	1.06
1994	1.09	55.96	1.33
1995	0.02	95.01	0.71
1996	-0.01	224.03	1.07
1997	1.24	81.78	0.89
1998	1.51	145.52	1.22
1999	1.55	101.50	0.98
2000	1.35	92.43	1.35
2001	1.49	167.66	1.26
2002	1.23	307.60	1.23

2003	1.18	190.10	1.66
2004	1.74	183.67	0.78
2005	0.97	275.30	1.28
2006	1.51	206.51	1.07
2007	2.08	117.96	0.58
2008	1.11	151.80	0.82
2009	1.89	70.19	0.67
2010	1.63	492.95	2.16

temperature					precipitation				
	spring	summer	autumn	winter		spring	summer	autumn	winter
1961	1.75	11.38	0.11	-13.02	1961	59.16	16.21	0.01	6.66
1962	1.97	10.44	-0.17	-11.01	1962	7.53	55.73	39.35	1.38
1963	2.72	10.91	1.50	-11.65	1963	20.13	59.78	1.32	25.63
1964	1.66	10.71	0.10	-12.96	1964	105.62	59.73	5.53	17.20
1965	1.90	10.62	1.25	-11.60	1965	35.46	87.53	6.17	7.42
1966	1.37	11.01	-0.35	-9.96	1966	21.37	73.52	41.10	32.70
1967	1.19	10.78	-0.25	-12.37	1967	36.16	30.37	2.50	0.21
1968	1.69	10.86	0.67	-11.29	1968	29.12	115.84	0.17	15.29
1969	2.40	10.84	1.01	-12.48	1969	27.28	60.51	0.05	7.85
1970	1.69	10.98	0.63	-9.89	1970	34.04	38.63	2.10	2.18
1971	2.80	11.30	0.18	-11.11	1971	29.04	72.88	30.04	28.85
1972	1.81	9.85	0.70	-11.85	1972	111.63	167.84	2.70	6.54
1973	2.32	12.04	1.15	-12.81	1973	30.84	79.65	3.74	39.58
1974	2.94	10.13	0.68	-14.10	1974	15.47	109.33	9.65	9.90
1975	1.81	11.00	0.83	-12.70	1975	3.60	43.72	52.59	28.75
1976	1.60	10.62	0.23	-10.84	1976	6.95	36.20	52.04	9.02
1977	2.43	11.62	2.04	-11.82	1977	7.34	99.98	48.76	47.63
1978	2.95	12.17	1.09	-14.26	1978	2.24	26.11	16.05	5.16
1979	1.38	11.05	0.91	-9.27	1979	12.84	59.64	1.74	19.46
1980	2.78	11.41	1.59	-11.70	1980	15.04	14.64	13.54	22.51
1981	3.55	10.35	-0.55	-10.86	1981	4.13	196.66	5.87	2.18
1982	1.98	10.41	0.30	-10.51	1982	47.12	116.32	24.88	10.46
1983	1.39	11.03	1.06	-12.33	1983	10.26	70.89	9.09	2.65
1984	2.17	11.99	0.13	-12.17	1984	17.30	33.36	12.77	6.07
1985	1.99	11.40	0.54	-10.90	1985	17.97	11.16	5.57	0.54
1986	0.77	11.07	0.64	-10.98	1986	7.15	37.36	2.39	12.73
1987	1.86	10.00	0.79	-9.63	1987	123.03	220.41	123.43	8.45
1988	1.28	10.81	0.57	-9.47	1988	120.63	105.55	6.24	1.03
1989	0.93	9.60	0.41	-11.10	1989	15.03	161.33	9.02	25.77
1990	1.99	11.48	1.26	-9.18	1990	29.18	43.28	0.09	13.56
1991	1.50	10.24	0.42	-11.26	1991	46.37	142.88	3.12	15.11
1992	1.39	10.25	0.15	-10.94	1992	40.45	124.53	12.60	28.02

1993	1.82	9.86	0.53	-10.21	1993	61.43	110.06	0.58	4.33
1994	1.79	12.04	0.84	-10.82	1994	3.63	38.46	4.39	12.60
1995	1.20	11.15	0.23	-12.07	1995	10.28	25.95	45.07	5.76
1996	0.68	9.83	0.62	-11.55	1996	56.72	158.00	3.77	0.44
1997	3.04	11.09	0.88	-8.93	1997	4.57	75.26	0.15	4.60
1998	2.36	10.90	1.93	-9.55	1998	18.88	93.58	13.51	6.78
1999	2.41	11.08	1.79	-9.65	1999	16.47	58.12	13.44	2.49
2000	3.32	11.15	0.99	-10.17	2000	9.62	67.78	8.73	0.93
2001	3.25	11.37	1.74	-10.48	2001	8.06	131.02	23.96	49.34
2002	3.37	11.47	1.43	-10.85	2002	10.46	231.04	45.09	13.34
2003	1.57	11.32	1.57	-9.24	2003	72.19	77.47	34.43	18.19
2004	3.50	11.08	1.70	-10.43	2004	72.63	65.00	12.10	20.83
2005	2.43	10.84	1.91	-10.91	2005	106.06	151.82	6.94	83.61
2006	3.19	11.57	3.16	-11.36	2006	11.86	117.42	1.53	0.19
2007	4.19	11.88	1.39	-9.04	2007	11.83	68.41	26.42	60.07
2008	4.10	12.28	1.87	-13.71	2008	37.05	29.81	13.74	1.34
2009	3.46	11.66	1.27	-9.23	2009	12.15	16.86	31.01	32.76
2010	3.07	11.12	1.88	-9.13	2010	79.47	332.85	67.79	5.44
			runoff						
				spring	summer	autumn	winter		
			1961	0.14	0.72	0.22	0.03		
			1962	0.12	0.93	0.11	0.03		
			1963	0.18	0.61	0.12	0.05		
			1964	0.09	1.13	0.12	0.04		
			1965	0.06	0.81	0.09	0.03		
			1966	0.08	1.14	0.30	0.04		
			1967	0.43	0.95	0.12	0.06		
			1968	0.21	0.86	0.12	0.05		
			1969	0.22	0.86	0.07	0.04		
			1970	0.07	0.83	0.19	0.05		
			1971	0.17	0.81	0.16	0.03		
			1972	0.16	0.98	0.16	0.05		
			1973	0.19	0.95	0.15	0.04		
			1974	0.09	1.16	0.13	0.03		
			1975	0.17	0.82	0.11	0.04		
			1976	0.10	0.86	0.05	0.06		
			1977	0.06	0.88	0.16	0.03		
			1978	0.17	0.92	0.07	0.04		
			1979	0.05	0.75	0.08	0.04		
			1980	0.13	0.67	0.09	0.03		
			1981	0.11	1.20	0.14	0.03		
			1982	0.21	1.10	0.13	0.03		

			1983	0.23	1.11	0.31	0.04		
			1984	0.11	0.85	0.09	0.04		
			1985	0.10	0.72	0.06	0.05		
			1986	0.07	1.15	0.08	0.03		
			1987	0.23	1.33	0.16	0.05		
			1988	0.29	1.20	0.15	0.08		
			1989	0.22	1.05	0.24	0.08		
			1990	0.21	0.95	0.17	0.08		
			1991	0.17	0.95	0.15	0.06		
			1992	0.08	0.89	0.17	0.07		
			1993	0.17	0.69	0.10	0.08		
			1994	0.09	0.99	0.15	0.06		
			1995	0.16	0.43	0.08	0.05		
			1996	0.10	0.88	0.05	0.02		
			1997	0.06	0.70	0.08	0.03		
			1998	0.07	0.90	0.18	0.04		
			1999	0.05	0.80	0.07	0.05		
			2000	0.16	1.07	0.07	0.03		
			2001	0.07	0.95	0.19	0.03		
			2002	0.10	0.90	0.16	0.04		
			2003	0.19	1.21	0.19	0.06		
			2004	0.09	0.56	0.06	0.06		
			2005	0.11	0.87	0.24	0.04		
			2006	0.11	0.80	0.10	0.04		
			2007	0.09	0.33	0.11	0.05		
			2008	0.10	0.63	0.05	0.03		

Variable	Period	N	U	H0	Abrupt change point
AN-AT	1961-1996	36	-5.24	R	1997
	1997-2010	14			
AN-AP	1961-1986	26	-2.36	R	1987
	1987-2010	24			
AN-AR	1961-1994	34	2.01	R	1995
	1995-2010	16			
SP-AT	1961-1996	36	-4.26	R	1997
	1997-2010	14			
SU-AT	1961-1996	36	-2.65	R	1997
	1997-2010	14			
AU-AT	1961-1996	36	-4.63	R	1997
	1997-2010	14			
WI-AT	1961-1996	36	-3.19	R	1997
	1997-2010	14			

SU-AP	1961-1986	26	-2.24	R	1987
	1987-2010	24			
SU-AR	1961-1994	34	2.12	R	1995
	1995-2010	16			

	The correlation relationship					
	Pearson correlation coefficients	AN-AR	SP-AR	SU-AR	AU-AR	WI-AR
	AN-AT	**-0.248				
	SP-AT		-0.281			
	SU-AT			**-0.337		
	AU-AT				-0.097	
	WI-AT					*0.335
	AN-AP	**0.545				
	SP-AP		*0.291			
	SU-AP			**0.403		
	AU-AP				0.087	
	WI-AP					0.046
	** means that correlation is significant at 0.01 level.					
	*. Correlation is significant at the 0.05 level					
	The path analysis					
		Path coefficient（direct）	Path coefficient（indirect）			
		P(MMT,MR)	MMT	MP		
MMT	Annual	**-0.333	_	**-0.024		
	Summer	**-0.362	_	**-0.112		
MP	Annual	**0.385	**0.021	_		
	Summer	**0.221	**0.185			
	** means that correlation is significant at 0.01 level.					
		P(MMT,MR)	MMT	MP		

MMT	Annual	**-0.280	_	**0.032		
MP	Annual	**0.561	**-0.016	_		
MMT	summer	**-0.366	_	**0.151		
MP	summer	**0.423	**-0.131	_		
			Indirect effect			
	Variable	Direct effect	AT	AP	Total	
	AN-AT	**-0.280		**0.032	**-0.296	
	AN-AP	**0.561	**-0.016		**0.593	
	SU-AT	**-0.366		**0.151	**-0.497	
	SU-AP	**0.423	**-0.131		**0.574	
